# Socket preservation utilizing polymeric bioresorbable membranes: a preclinical model

**DOI:** 10.4317/medoral.26938

**Published:** 2025-02-15

**Authors:** Edisa Oliveira Sousa, Nicholas A Mirsky, Marcelo Parra, Vasudev Vivekanand Nayak, Bruno Luís Graciliano Silva, Estevam A Bonfante, Nick Tovar, Paulo G Coelho, Lukasz Witek

**Affiliations:** 1 Department of Prosthodontics and Periodontology, Bauru School of Dentistry, University of Sao Paulo, Bauru, Brazil; 2 University of Miami Miller School of Medicine, Miami, Florida, USA; 3 Department of Comprehensive Adult Dentistry, Faculty of Dentistry, Universidad de la Frontera, Temuco, Chile; Center of Excellence in Morphological and Surgical Studies (CEMyQ), Faculty of Medicine, University of La Frontera, Temuco, Chile; 4 Department of Biochemistry and Molecular Biology, University of Miami Miller School of Medicine, Miami, Florida, USA; 5 Department of Diagnosis and Surgery, School of Dentistry of Araraquara, São Paulo State University (UNESP), Araraquara, Brazil; 6 Private Practice, Boynton Beach, FL, USA; 7 DeWitt Daughtry Family Department of Surgery, Division of Plastic and Reconstructive Surgery, University of Miami Miller School of Medicine, Miami, Florida, USA; Department of Biochemistry and Molecular Biology, University of Miami Miller School of Medicine, Miami, Florida, USA; 8 Biomaterials Division, NYU Dentistry, New York, NY USA; Hansjörg Wyss Department of Plastic Surgery, NYU Grossman School of Medicine, New York, New York, USA; Department of Biomedical Engineering, NYU Tandon School of Engineering, Brooklyn, New York, USA

## Abstract

**Background:**

The preservation of the alveolar ridge following tooth extraction is crucial to prevent atrophy and maintain structural integrity, facilitating future dental rehabilitations. This study compared the use of two different polymeric, resorbable membranes: polylactic acid (PLA), and 5% polylactic acid + 95% polycaprolactone (PLA/PCL), relative to unassisted socket healing (negative control).

**Material and Methods:**

A preclinical model involving healthy, skeletally mature beagles (*n*=7) were used in this study. Surface topography and thermal degradation of the membranes were assessed, followed by *in vivo* evaluation of socket preservation in extracted maxillary premolars. Histomorphometric analysis was employed to measure bone formation and total socket area. Data was analyzed through linear mixed models with fixed factor of treatment following a post-hoc comparison by the Tukey test. Ranked data of residual membrane presence and inflammatory infiltrate were analyzed through Kruskal-Wallis non-parametric test. All analyses were conducted with statistical significance set at *p-value* ≤ 0.05.

**Results:**

Surface topography depicted a distinctive fibrous network structure for PLA membrane relative to PLA/PCL which exhibited a more porous architecture. Thermal degradation behavior/profile, observed through TGA and DSC, for both membranes was similar. Histomorphometric analysis of bone formation within the induced socket yielded 36.1 ±7.7%, 35.6 ±7.2% and 32.8 ±7.7% for control, PLA and PLA/PCL groups, respectively, with no statistically significant differences between groups (*p* = 0.796). Analysis of total socket area (mean ± 95% confidence intervals) yielded significantly higher values for experimental groups, PLA (8.95 ± 1.7 mm2) and PLA/PCL (8.8 ± 1.76 mm2), relative to control (6.7 ± 1.8 mm2) (*p* = 0.041). Residual membrane, along with mild inflammatory infiltrate was observed after the healing period irrespective of membrane type utilized.

**Conclusions:**

Guided bone regeneration (GBR) with PLA and PLA/PCL membranes did not yield higher bone formation within the socket relative to the control group. However, an improvement in the preservation of the socket’s architecture was observed.

** Key words:**Alveolar ridge preservation, resorbable membranes, polylactic acid, polycaprolactone, guided bone regeneration.

## Introduction

The alveolar bone is a complex structure whose primary roles are to support tooth roots, tightly joined by connective tissue fibers known as the periodontal ligament (PDL), and to distribute forces generated during oral functions (1). Alveolar bone is comprised of two components: the alveolar bone proper, a dense cortical plate housing the tooth socket and anchoring the PDL, and the supporting alveolar bone - comprising of cortical plates and trabecular bone which extends from the alveolar ridge to provide external coverage to the alveolar process (1,2). The alveolar bone is recognized as a tooth-dependent structure that develops concurrently with tooth eruption and undergoes atrophy and morphological changes following tooth loss (1,2). Following tooth extraction, structural reconFiguration occurs through a sequence of remodeling steps within the surrounding hard and soft tissues, eventually leading to atrophy at the affected sites (3-5). Alveolar ridge atrophy represents a progressive, and irreversible phenomenon that gives rise to numerous surgical, prosthetic, esthetic, and functional challenges within the field of oral rehabilitation (6). Numerous *in vivo* studies evaluating tooth extraction have described in detail the alveolar atrophy, and bone resorption process (4,6-8).

Bone resorption following tooth loss has been described to be comprised of two clinically distinct phases - (i) an initial healing phase of rapid resorption (~6 months) with a peak activity at 3-4 weeks ; and (ii) a subsequent, gradual resorptive phase that persists indefinitely (9). Furthermore, it has been observed that the most substantial bone loss occurs horizontally rather than vertically (10). Quantitatively, ~60% of alveolar bone width, and ~40% of alveolar bone height are lost within the first six months following tooth extraction, which corresponds to an average loss of ~3-4 mm in width and ~1.5-2.0 mm in height of bone tissue (3,11-13).

The alveolar ridge assisted healing concept was proposed to prevent alveolar ridge reabsorption, as preservation has been shown to facilitate bone formation within the socket (12,14). Moreover, preserving an adequate three-dimensional osseous volume within an extraction site directly impacts the success of dental implants rehabilitations by ensuring a biomechanical competence (8,12,15). Techniques that support alveolar ridge preservation include immediate socket grafting using particulate bone grafts or substitutes, and guided bone regeneration (GBR) through membrane coverage (12).

Previous investigations into alveolar ridge preservation techniques involving graft materials and/or membranes have consistently demonstrated noTable advantages (7,11,12,16). These benefits include reduced post-extraction bone loss, preserved ridge volume, and adequate soft tissue volume (12). Alveolar ridge preservation through GBR relies upon specific fundamental requirements. First, it demands defect site occlusivity to effectively prevent the invasion of epithelial and connective tissues, which may otherwise proliferate within the defect site and impede desired bone tissue regeneration. Second, it necessitates mechanical stability of the membrane, in order to maintain a space for proliferating osteogenic cells to migrate into the defect site (17). Furthermore, ideal membranes must be biocompatible, non-immunogenic, and non-toxic. In the context of these requirements, bioresorbable membranes have emerged as an excellent choice due to their ability to naturally dissolve, eliminating the need for post-healing membrane retrieval (18). This not only minimizes potential morbidity but also reduces the time and cost of care (17).

Synthetic resorbable materials, such as polylactic acid (PLA) and polycaprolactone (PCL), have been previously considered for GBR application due to their advantageous properties. These include low rigidity, ease of trans-operatory handling, processability, biodegradability, and their ability to support cellular attachment, differentiation, and proliferation (17,19,20). PLA membranes consist of a unique, porous three-layer technology which have shown successful molecular weight (MW) reduction after ~5 months of implantation, ultimately resulting in complete resorption within one year timeframe (20). In contrast, PCL membranes are distinguished by higher hydrophobicity and lower water solubility compared to PLA membranes. These properties may contribute to PCL having lower cell affinity and greater biodegradation time, which exceeds 12 months (17,18). Studies regarding combined PLA and PCL (PLA/PCL) polymer membranes have demonstrated suiTable biological requirements, although a longer degradation process is seen when compared to pure PLA membranes (21). Abe *et al*., (22) conducted an *in vitro* study assessing the physicochemical and biological properties of the combined PLA/PCL polymer membrane when applied to GBR. The results revealed that the experimental membrane exhibited a high biocompatibility and slow degradation rate, with nearly half of the material still present after 26-weeks of observation. Further, PLA/PCL membranes have shown promising ridge preservation application, demonstrating less resorption in ridge width and height when compared to an unassisted socket defect (11,16). Nevertheless, there remains no consensus regarding the utilization of these resorbable membranes in the healing process of extraction sockets.

The purpose of the current, pre-clinical, *in vivo* study was to compare the effectiveness of a well-established PLA membrane and the combined PLA/PCL resorbable membrane to an unassisted socket defect. The specific aim of the study was to compare effectiveness of treatment by quantitatively and qualitatively analyzing bone formation within the socket and socket total area. The null hypotheses of the present study were: (i) the use of resorbable membranes and their variable composition would not significantly impact bone regeneration, and (ii) the use of resorbable membranes and their variable composition would not significantly influence alveolar ridge preservation.

Materials and Methods

- Study Design

Material Characterization: Scanning electron microscopy (SEM) (Zeiss EV-50, Oberkochen, Germany) was utilized to analyze the surface topography of the membranes. Imaging was performed at 5 kV, under a current of 2.5 nA to observe surface topographies of the polymeric experimental groups. Differential scanning calorimetry (DSC) and thermogravimetric analysis (TGA) were conducted for the two membranes using a DSC/TGA system (SDT Q600, TA Instruments-Waters, LLC, New Castle, DE). Weight change (%) and heat flow (mV) data was collected as a function of temperature (°C). Approximately 10 mg of the sample (PLA or PLA/PCL) was placed in the heating chamber of the instrument and the temperature was ramped linearly at a rate of 20°C/min from 25 to 1000°C in the presence of Argon gas. Data obtained was analyzed using the TA Universal Analysis 2000 software package (TA Instruments-Waters, LLC, New Castle, DE).

Preclinical In Vivo Model: The study was approved by the bioethics committee and institutional review board (IRB) for animal experimentation from École nationale vétérinaire d'Alfort (EnvA), Maisons-Alfort, France and consisted of seven adult, skeletally mature, beagle dogs (aged ~1.5 years) in good health. The subjects were allowed to acclimate at the facility for 7 days before surgery. Prior to the surgical procedure, animals were fasted for at least 12 hours, following which they were anesthetized with an intramuscular injection of atropine sulfate (0.044 mg/kg) and xylazine chlorate (8 mg/kg). General anesthesia was then administered with an intramuscular injection of ketamine chlorate (15 mg/kg). Subsequently, the bilateral maxillary premolars (2nd and 3rd) were atraumatically extracted to prevent damage to the alveolar bone wall. In brief, a full thickness mucoperiosteal flap was raised, and the teeth were extracted by sectioning the roots in the buccolingual direction.

After extraction, the sockets were treated as follows in a randomized fashion: (i) no membrane, as a control group; (ii) poly(lactic acid) membrane (PLA) (Epi-guide, THM Biochemical Inc., Duluth, MN, USA); and (iii) 5% PLA + PCL - poly(caprolactone) membrane (PLA/PCL) (DSM Biomedical, Exton, PA, USA). Membranes were trimmed to ensure complete coverage of both sockets, and immediately sutured with 4-0 resorbable sutures on both sides of the maxilla. To prevent postoperative infections, antibiotics (penicillin, 20,000 UI/kg) and were provided for a period of 48 hours. Postoperative analgesics (ketoprofen, 1 mL/5 kg) were also administered as required, and food and water were provided *ad libitum*. All animals were sacrificed after 6 weeks following surgery by means of anesthesia overdose. Mandibles were harvested by sharp dissection and immediately fixed in 10% formalin prior to further processing.

- Histologic Preparation and Histomorphometric Analysis

Specimens were gradually dehydrated in a series of ethyl alcohol solutions ranging from 70% to 100%. Following dehydration, the samples were embedded in a methacrylate-based resin (Technovit 9100; Heraeus Kulzer, Wehrheim, Germany) per the manufacturer’s instructions. The blocks were then cut in a bucco-palatal direction, following root long-axis inclination, into slices (~300 μm thickness) with a precision diamond saw (Isomet 2000; Buehler, Lake Bluff, IL). The slices were then glued to acrylic slides using an acrylate-based glue and allowed to set for 24 hours prior to grinding and polishing. The sections were reduced to a final thickness of ~100 μm by means of a series of silicon carbide (SiC) abrasive papers (600, 800, and 1200 grit) utilizing a grinding/polishing machine (Metaserv 3000, Buehler, Lake Bluff, IL) under continuous water irrigation. Subsequently, the samples were stained with Stevenel’s Blue and Van Giesons’s Picro Fuschin (SVG) and digitally scanned using an automated slide scanning system equipped with a specialized computer software (Aperio Technologies, Vista, CA, USA) (23). Stevenel’s Blue stained cells and extracellular structures in a subtle gradation of blue tones. The counterstain, Van Gieson’s picro-fuchsin, stained collagen fibers in green or green-blue; bone in orange or purple; osteoid in yellow-green; and muscle fibers in blue to blue-green. This staining combination permitted for the differentiation between the soft, connective, osteoid, and mineralized tissues.

- Variables

Exposure/Independent Variable: The study investigated the application of PLA and PLA/PCL membranes compared to control (no membrane application).

Main Outcome Variable(s): Primary outcomes included the quantification of bone formation and the total socket area. Secondary outcomes involved the assessment of residual membrane presence and inflammatory infiltrate.

- Data Collection Methods

Histological observation and histomorphometric evaluations were conducted whereby the amount of bone formation within socket (%), socket total area (mm2) were quantified by means of a computer software (Image J; National Institutes of Health, Bethesda, MD Leica Application Suite, Leica Microsystems) (24).

Membrane presence and inflammatory infiltrate content were ranked, by a single blinded and trained histopathologist using the histomicrographs. Visual observation was performed along the cross-section of the bucco-palatal alveolar process using a 0-5 scale, as follows : (i) Membrane Presence: A rank of 5 constituted entire coverage of the surgical site with visible presence of the membrane, while a rank of 0 denoted fibrous structure at the surgical site with no visible presence of the membrane (23); (ii) Inflammation: For inflammatory cells content at the surgical site and/or membrane/host tissue interface, a rank of 0 indicated no inflammation, while a rank of 5 implied a significant inflammation (23).

- Statistical Analyses

Preliminary analyses of amount of bone formation within socket and socket total area showed indistinguishable variances in the study of the dependent variable (Levene test, *p* > 0.25). Data were collected and statistically evaluated through linear mixed models with fixed factor of treatment (Control, PLA and PLA/PCL) following a post-hoc comparison by the Tukey test, and data presented as the mean values with the corresponding 95% confidence interval (CI) values. Ranked data of residual membrane presence and inflammatory infiltrate were analyzed through Kruskal-Wallis non-parametric test. The data are presented as medians and interquartile ranges (IQR). All analyses were accomplished using SPSS (IBM SPSS v29, IBM Corp., Armonk, NY).

## Results

**Figure 1:**
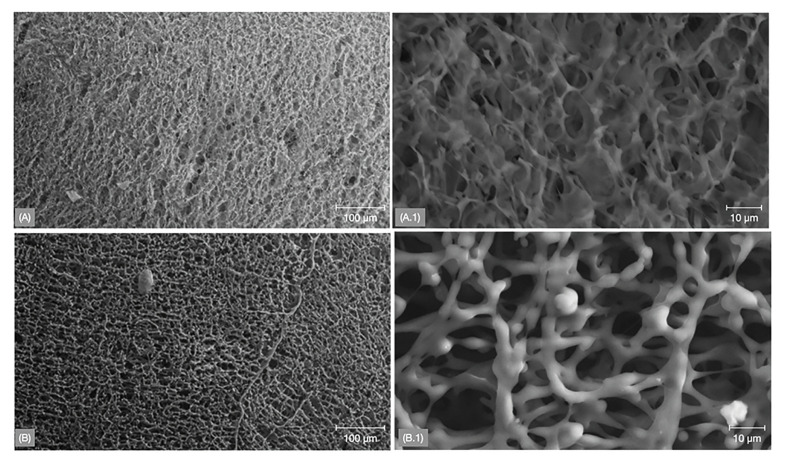
Scanning electron microscope (SEM) images of PLA (A and A.1) and PLA/PCL (B and B.1) membrane groups. A fibrous network structure can be evidenced for both group with noticeable more porous arrangement for PLA/PCL group.

**Figure 2:**
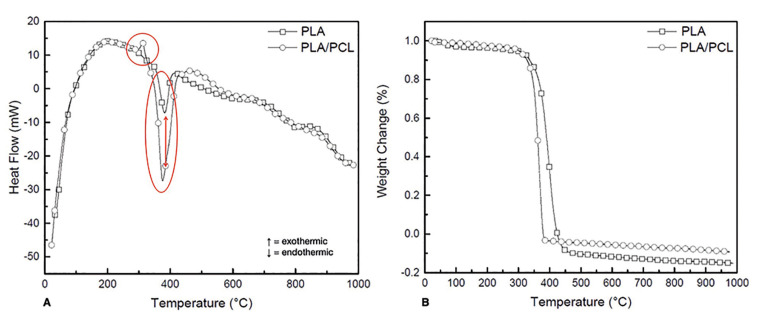
A. Differential scanning calorimetric (DSC) curves for PLA and PLA/PCL membranes evidencing similar transition temperatures for both groups, however, a higher enthalpy of transition is required for PLA/PCL. B. Thermogravimetrical (TGA) curves for PLA and PLA/PCL membranes demonstrating the similar thermal degradation behavior.

Scanning electron microscopy (SEM) micrographs depicted a distinct fibrous network topography between PLA and PLA/PCL (Fig. 1) membranes, with the latter exhibiting a more porous arrangement. Differential scanning calorimetric (DSC) analysis revealed that both membranes exhibited a similar transition temperature (~350°C), however, a higher amount of heat (enthalpy of transition) was required for PLA/PCL relative to the PLA membrane (Fig. 2). Additionally, thermogravimetric analysis (TGA) demonstrated similar weight loss (%), with an overlap in the onset point and derivative weight loss peak temperature between both membranes - indicating similar thermal degradation (Fig. 2).

**Figure 3:**
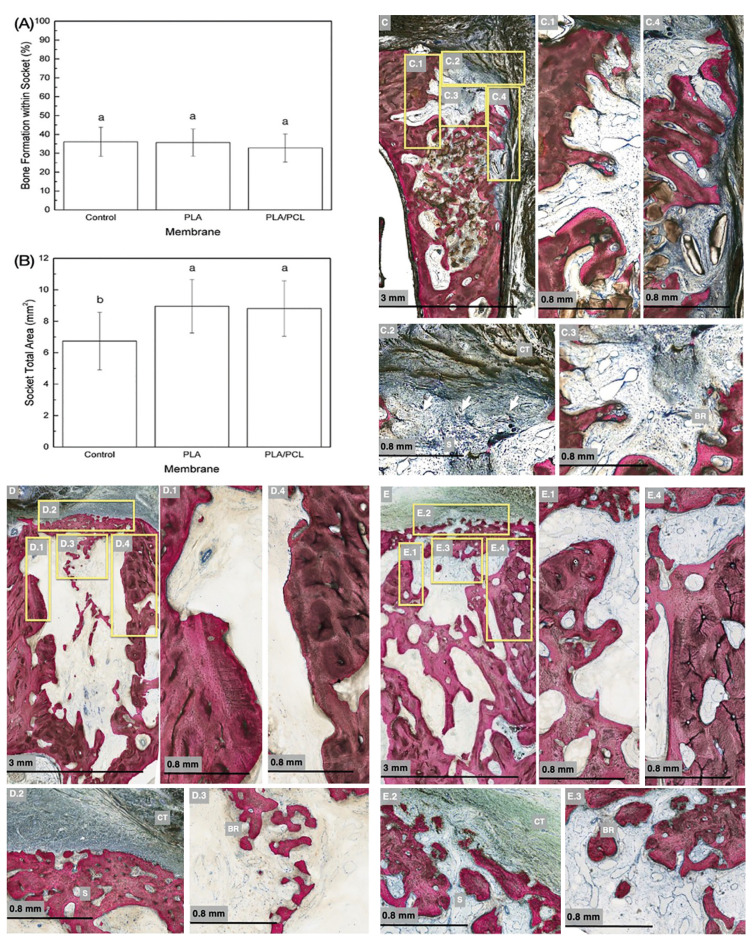
(A) Percentage of bone formation within socket as a function of treatment. Similar amount of new bone among groups was evidenced. Data presented as mean and 95%CI. Identical letters indicate no significant difference among groups; (b) Socket total area, in mm2, as a function of treatment evidencing significant higher values when membranes were used. Data presented as mean and 95%CI. Identical letters indicate no significant difference among groups; (C) Histological micrographs of Control group. (C.1) High magnification of palatal plate showing remodeling process. (C.2) Occlusal portion of the socket (S) with soft tissue infiltration evidence (arrows), presence of inflammatory infiltrate content and connective tissue (CT). (C.3) High magnification of the central part of the socket with bone remodeling (BR). (C.4) High magnification of the buccal plate with evident bone remodeling; (D) Histological micrographs of PLA group. (D.1) High magnification of palatal plate showing remodeling process. (D.2) Occlusal portion of the socket (S) with no soft tissue infiltration evidence and remodeling membrane/connective tissue interface (CT). (D.3) High magnification of the central part of the socket with bone remodeling (BR). (D.4) Closer view of the buccal plate also evidencing the remodeling process; (E) Histological micrographs of PLA/PCL group. (E.1) High magnification of palatal plate showing remodeling process. (E.2) Occlusal portion of the socket (S) with no soft tissue infiltration evidence and remodeling membrane/connective tissue interface (CT). (E.3) High magnification of the central part of the socket with bone remodeling (BR). (E.4) High magnification of the buccal plate also evidencing the remodeling process.

Surgical interventions demonstrated no complications regarding procedures, postoperative infections and/or other clinical concerns. No adverse events, such as membrane exposure, were detected, and clinically healthy soft tissue was observed at the surgical site throughout the course of the study during follow ups. The percentage of bone (mean ± 95% CI) formation within the socket after 6 weeks showed 36.08 ±7.73%, 35.65 ±7.20% and 32.82 ±7.74% for control group, PLA and PLA/PCL groups, respectively, with no significant differences (*p*>0.79) (Fig. 3). Socket total area (mean ± 95% CI) was significantly higher for groups PLA (8.95±1.7 mm2) and PLA/PCL (8.8 ±1.7 mm2), when compared to control group (6.7 ±1.8 mm2) (*p*=0.041). No significant differences in socket area were identified between PLA and PLA/PCL membranes (*p*=0.992) (Fig. 3).

Representative micrographs for control, PLA, and PLA/PCL groups are presented in Fig. 3, respectively. Qualitative histological assessments revealed soft tissue infiltration within the sockets in the control group. Higher magnification histomicrographs demonstrated the bone remodeling process, particularly evident in the occlusal section of the socket, which exhibited soft tissue invagination along with inflammatory infiltrate and connective tissue. In contrast, the membrane-covered groups (PLA and PLA/PCL) displayed no indications of soft tissue infiltration within the sockets, with the central aspect indicating bone remodeling. Furthermore, bone formation occurred in the most cervical region of the alveolus, while the unassisted socket group exhibited more pronounced bone loss on the buccal plate, both in thickness and height. This resulted in a significantly smaller socket total area, as confirmed by histomorphometric analysis.

**Figure 4:**
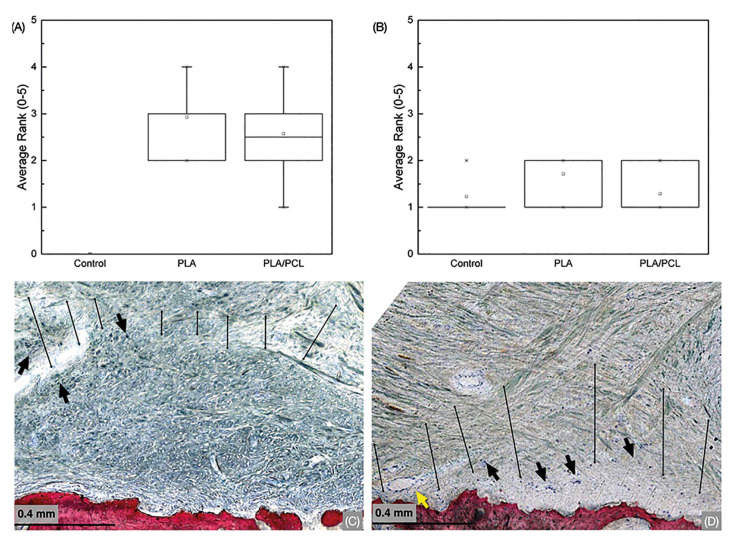
(A) Presence of residual membrane after 6-week healing evidencing no significant difference between PLA and PLA/PCL and (B) Mild inflammatory infiltrate content for all groups was observed. Data presented as median and quartiles. Letters statistically homogenous groups. High magnification micrographs of PLA (C) and PLA/PCL (D) groups evidencing residual membrane presence (within marked lines) and its resorption process through the existence of inflammatory infiltrate cells (black arrows). A blood vessel is depicted within the membrane structure (yellow arrow).

The quantification and ranking of residual membrane presence revealed no significant differences between membrane types (*p*>0.28) (Fig. 4). PLA and PLA/PCL groups exhibited residual membrane presence and uneven, soft tissue membrane remodeling at 6 weeks *in vivo* at higher magnifications. Both membrane treatment groups revealed connective tissue development and blood vessel infiltration in the region of interest (Fig. 4). Furthermore, histological observations indicated mild levels of inflammatory infiltrate, concentrated in regions near the membranes for both groups (Fig. 4). Notably, there was no significant difference among groups concerning the ranked inflammatory infiltrated content (*p*>0.3) (Fig. 4).

## Discussion

Successful rehabilitation of atrophic edentulous areas depends not only on the physiological outcomes of the defect but also on meeting the aesthetic demands of patients, posing a challenge for clinicians (8,25). Maintaining an adequate three-dimensional ridge volume, including a buccal plate with sufficient thickness and height is a fundamental prerequisite for achieving esthetically pleasing rehabilitations (8,12,15). Furthermore, preservation of alveolar ridge volume plays a critical role in averting biomechanical complications and potential failures of subsequent treatments after GBR (8). The dual-directional alveolar resorption process results in narrower ridges with reduced height. Moreover, a greater resorption on the buccal side may lead to a lingual/palatal shift of the long axis (2,4). All previously mentioned changes in unassisted socket healing potentially limit the execution of an effective prosthetic treatment with adequate functional and esthetic outcomes (2,4).

Alveolar ridge preservation outcomes have been aided by GBR techniques, the primary role of which is to provide a favorable space for bone formation by utilizing barrier membranes. The establishment of a conducive environment for osteogenic cell-facilitated bone regeneration is achieved through the isolation of the blood clot within the defect, effectively preventing epithelial and connective tissue invasion (11). With the objective of evaluating the healing process of extraction sockets, this study conducted a comparative analysis, examining the use of two distinct polymeric resorbable membranes, namely PLA and PLA/PCL, in comparison to an unassisted socket (control group).

Although both PCL and PLA are linear aliphatic polyesters, they exhibit distinct molecular structures. PCL, is characterized by its robust, hydrophobic, and crystalline nature, featuring slower degradation kinetics compared to PLA. In contrast, PLA is inherently stiffer and tougher than PCL. The blending of PLA and PCL allows for the preservation of the respective advantages of each polymer, while simultaneously mitigating some of their individual drawbacks . However, in the current study, no significant differences in ridge architecture preservation between both types of membranes was observed. This observation concurs with the findings of Rowe *et al*., (26) who reported homogeneous cellular proliferative capacities across pure PCL, pure PLA, and PLA/PCL membranes. This uniformity in behavior may be attributed to the minimal presence of PLA within the PLA/PCL membranes (5%), resulting in analogous *in vivo* outcomes. Nonetheless, additional research is imperative to ascertain the optimal polymer concentrations.

This study demonstrates that adopting alveolar ridge preservation techniques through resorbable membrane application offers the possibility to maintain the dimensions of the alveolar crest, or at least reduce its modifications. Socket total area (mm2) exhibited a significant increase when membrane coverage was applied, irrespective of its chemical composition. This reasoning relies on the assumption that the primary purpose of the membrane is to provide the necessary space for bone formation, thereby contributing to a greater area available for regeneration (27). Systematic reviews have confirmed this beneficial clinical outcome of socket area preservation using GBR techniques (28,29). In contrast, the current study showed that the control group exhibited greater resorption of the buccal bone plate coupled with infiltration by soft tissue into the alveolus. Previous studies investigating soft tissue invagination appear to support the positive clinical outcomes of isolating socket sites by utilizing resorbable membranes in GBR (28,29).

From a thermal stability perspective, both PLA and PLA/PCL membranes demonstrated similar thermal degradation behavior and were sTable at physiological temperatures. The mechanism of biodegradation of lactic acid-based polymers, and several copolymers have been detailed to be qualitatively similar, despite structural and morphological differences (30). To elaborate, the initiation of the degradation process has been detailed to be non-enzymatic random hydrolytic ester cleavage, the duration of which is determined by the initial molecular weight of the polymer and the chemical structure. The *in vivo* data regarding the ranking of residual membrane presence showed no significant differences between both membrane compositions. This observation aligns with the *in vitro* findings of Abe *et al*., (22) where a PLA/PCL membrane performed comparably to a poly(lactic-glycolic-acid) (PLGA) membrane after 6 weeks of assessment.

Furthermore, in accordance with Lekovic *et al*., (11) it is crucial for membranes to remain in place for 4 to 6 weeks to achieve maximum regenerative results during which membrane degradation processes occur. Such processes pertaining to the use of lactic acid-based membranes are often accompanied by an inflammatory reaction, creating an acidic environment, which is not ideal for early tissue development (21). Although lactic acid-based membranes are non-cytotoxic and biodegradable, the releases of oligomers and acid byproducts during degradation may trigger inflammation reactions and foreign body response *in vivo* . Upon closer examination at higher magnifications, a mild inflammatory infiltrate content was observed in the membrane-soft tissue interface for both experimental groups, with no significant differences between them. However, this level of inflammation was not detrimental to bone healing within the socket, as statistically similar bone formation was observed in the control group.

Concurrently with these findings there is a discernible surge in interest among clinicians surrounding various guided bone regeneration (GBR) techniques. As previously established, the clinical rationale for alveolar ridge preservation is to mitigate the need for future interventions focused on bone reconstruction. Maintaining an ideal bone architecture allows for a more conservative, predicTable, and prognostically advantageous rehabilitation. Additionally, the combination of membrane placement with a socket grafting material has demonstrated improved bone regeneration and alveolar ridge preservation (28); although further studies are warranted.

## Conclusions

Although GBR with PLA and PLA/PCL membranes did not increase the amount of bone formation within socket relative to control, it significantly improved the preservation of the socket architecture as demonstrated by socket total area. Additionally, the membrane type had no influence on alveolar ridge preservation outcomes and inflammation levels.
